# A regularly irregular rhythm—what is the diagnosis?

**DOI:** 10.1007/s12471-015-0787-1

**Published:** 2015-12-21

**Authors:** A-D. Margulescu, O.A. Enescu, D. Vinereanu

**Affiliations:** 1 University of Medicine and Pharmacy Carol Davila, Bucharest, Romania; 2 Department of Cardiology, University and Emergency Hospital of Bucharest, Bucharest, Romania

**Fig. 1:**
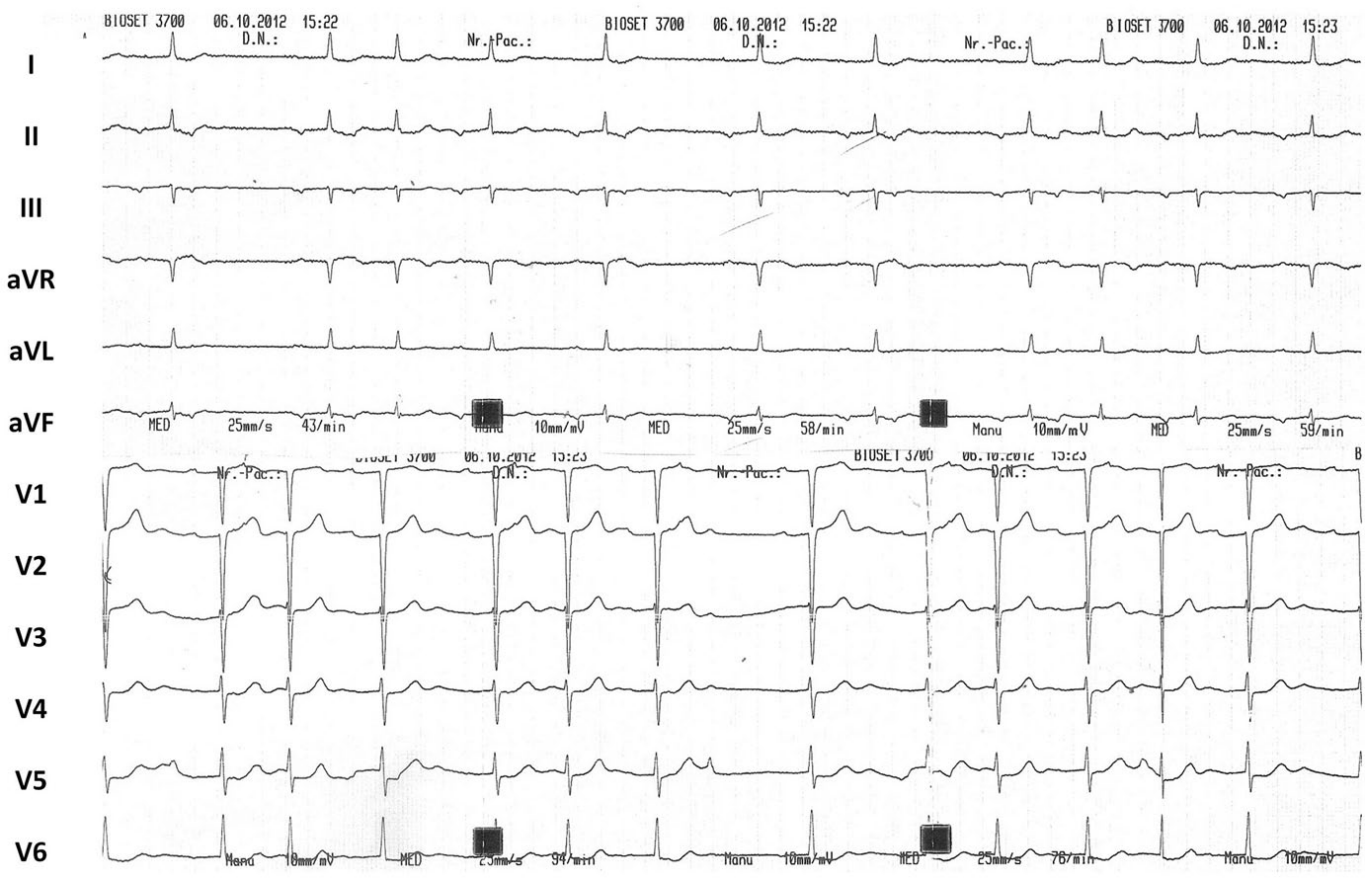
Twelve-lead ECG at admission

A 58-year-old woman was admitted with a 2-week history of dizziness and headache. She reported no syncope, chest pain or dyspnoea. Her medical history included hypertension and hypercholesterolaemia for which she was not taking any medications. She had stopped smoking 5 years earlier, and did not drink alcohol or use illicit drugs. On admission, her blood pressure was 200/100 mmHg, and the pulse was regularly irregular with a mean rate of 80 beats/min. The rest of the clinical examination was unremarkable. Except for a moderately increased erythrocyte sedimentation rate, all other laboratory results were normal. The chest X-ray was also normal. The twelve-lead ECG is displayed in Fig. 1.

What does the ECG show? Is this only a rhythm problem or should other diseases be sought? How should the patient be managed?

**Answer**

You will find the answer elsewhere in this issue.

